# Rationally engineered PEGylated l-citrulline functionalized baicalein encapsulated HSA nanopolymer guided by molecular docking for tumor microenvironment responsive and redox modulated colon cancer therapy

**DOI:** 10.1039/d5ra08629a

**Published:** 2026-01-02

**Authors:** Sounik Manna, Suman Mondal, Angsuman Das Chaudhuri, Gouri Karan, Surya Kanta Dey, Dibyendu Giri, Malay Dolai, Avijit Kumar Das, Sujata Maiti Choudhury

**Affiliations:** a Cancer Nanotherapeutics and Toxicology Research Laboratory, Biochemistry, Molecular Endocrinology, and Reproductive Physiology Division, Department of Human Physiology, Vidyasagar University Midnapore-721102 West Bengal India sujata_vu@mail.vidyasagar.ac.in sujata.vu2009@gmail.com; b Department of Allied Health Sciences, Brainware University Barasat Kolkata 700125 West Bengal India; c Department of Chemistry, Prabhat Kumar College Purba Medinipur 721404 West Bengal India; d Department of Chemistry, Christ University Hosur Road Bangalore Karnataka 560029 India avijitkumar.das@christuniversity.in

## Abstract

Colon cancer remains a major global health burden characterized by uncontrolled proliferation, oxidative stress, and poor responsiveness to conventional therapies, underscoring the need for biocompatible and targeted nanotherapeutic interventions. In this study, a novel pH-responsive human serum albumin-based nanocarrier, HSA-BA@PEG-LC NPs, was designed for the efficient and selective delivery of baicalein (BA) to colon cancer cells. Molecular docking analysis demonstrated strong binding affinities of BA with Hsp90 inhibitors and with human serum albumin (HSA), as well as a notable interaction between l-citrulline (LC) and the cationic amino acid transporter 1 (CAT-1), highlighting their potential roles in anticancer modulation. The engineered nanoparticles exhibited a uniform spherical morphology (232 nm), low polydispersity index (PDI < 0.3), and high colloidal stability (−27.21 mV). Spectroscopic analyses (FTIR and ^1^H NMR) confirmed successful encapsulation of BA and PEG-LC surface conjugation, with an encapsulation efficiency of 86.62% and pH-dependent sustained release favoring acidic tumor conditions. In HCT-116 cells, HSA-BA@PEG-LC NPs demonstrated enhanced internalization, strong cytoplasmic accumulation, and pronounced cytotoxicity (IC_50_ = 5.42 µg mL^−1^), while maintaining safety toward normal lymphocytes. Mechanistically, treatment induced elevated ROS levels, GSH depletion, mitochondrial depolarization, nuclear condensation, cytoskeletal collapse, and G0/G1 cell-cycle arrest. Furthermore, the formulation displayed potent antioxidant activity across DPPH, NO, SOD, and lipid peroxidation assays, with IC_50_ values approaching ascorbic acid, validating synergistic PEG-LC functionalization and HSA-mediated stabilization as a promising redox-driven nanoplatform for targeted colon cancer therapy.

## Abstract 2

Colon cancer remains a major global health burden characterized by uncontrolled proliferation, oxidative stress, and poor responsiveness to conventional therapies, underscoring the need for biocompatible and targeted nanotherapeutic interventions.

## Graphical abstract

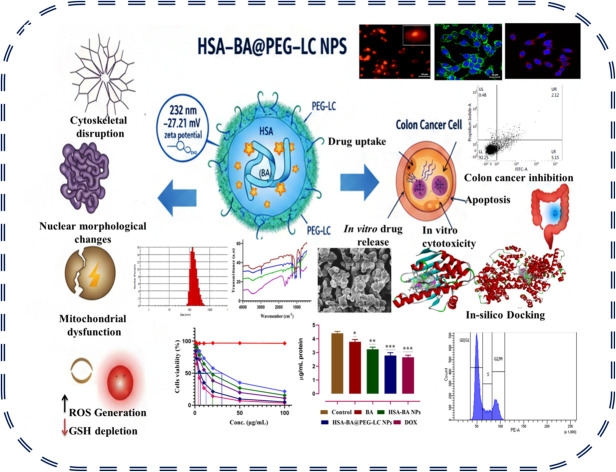

## 1 Introduction

Cancer continues to be one of the most pressing global health challenges, accounting for millions of deaths each year despite significant advancements in diagnostic and therapeutic modalities. The incidence and mortality rates of various cancers continue to raise worldwide, with cancer being the most important cause of morbidity and mortality across populations.^1^ Among malignancies, colorectal cancer (CRC) stands out as one of the most aggressive and fatal types, particularly affecting older adults. According to data from the Global Cancer Observatory (GLOBOCAN 2020), colorectal cancer (CRC) represents about 10% of all newly diagnosed cancer cases worldwide and contributes to roughly 9.4% of cancer-related deaths. This makes CRC the third most commonly trendy cancer and subsequent one foremost cancer mortality globally.^2^ The growing prevalence of CRC has been attributed to multiple factors, including aging populations, sedentary lifestyles, dietary patterns, and genetic predispositions. Despite progress in conventional treatment modalities such as surgery, chemotherapy, radiotherapy, and targeted therapies, the overall survival rate remains unsatisfactory. The emergence of therapeutic resistance, tumor heterogeneity, and severe off-target toxicities significantly limit the success of current regimens.^3^ Hence, there is crucial to develop novel and effective therapeutic strategies that improve treatment efficacy while minimizing adverse effects.

In current years, there has been rising attention to integrating natural bioactive compounds with advanced nanotechnology-based drug delivery systems to enhance cancer treatment outcomes. Natural phytochemicals, especially flavonoids, have gained considerable attention for their broad-spectrum pharmacological properties, including anticancer, antioxidant, and anti-inflammatory effects.^4^ Among these, baicalein (5,6,7-trihydroxyflavone) is a naturally occurring flavonoid isolated predominantly from the roots of *Scutellaria baicalensis*. Chemically, it belongs to the flavone subclass and consists of a three-ring phenolic structure with hydroxyl groups at positions 5, 6, and 7 on the A-ring, which confer strong antioxidant, redox-modulatory, and reactive oxygen species (ROS) scavenging properties.^4^ Baicalein stands out among natural flavonoids such as quercetin or resveratrol due to its relatively superior gastrointestinal permeability and distinct metabolic profile. Unlike highly polar glucuronides (*e.g.*, baicalin), baicalein lipophilic aglycone moiety facilitates absorption, enabling better systemic exposure. Although its aqueous solubility is modest (0.052 mg mL^−1^ in water), oral bioavailability in non-human primate studies reaches 13–23%, which compares favourably to many flavonoids with negligible absorption. Once absorbed, however, baicalein is rapidly conjugated: in rats 75.7% of an intravenous dose circulates as glucuronide/sulfate metabolites, and after oral dosing only conjugates are detectable in plasma; estimated absorption is 40% relative to dose administered intravenously. This combination moderate solubility, reasonable oral bioavailability, and efficient metabolic transformation support baicalein unicity among natural flavonoids and validates its selection for further pharmacological development.^5^ In recent years, baicalein has attracted considerable attention in oncology due to its broad spectrum of anticancer activities, including the ability to induce apoptosis, inhibit proliferation, and modulate multiple signaling pathways (*e.g.*, PI3K/Akt, NF-κB, and Wnt/β-catenin). These pathways are particularly relevant in colorectal carcinogenesis. Moreover, baicalein exhibits preferential cytotoxicity toward colon cancer cells, suppresses tumor angiogenesis, and has been shown to attenuate inflammatory components of the tumor microenvironment, which are key drivers of colon tumor progression. Its intrinsic redox-active nature and compatibility with hydrophobic pockets of carrier proteins such as HSA make baicalein an excellent candidate for encapsulation and nanocarrier engineering. Therefore, we selected baicalein not only as a therapeutic payload but also as a functional core molecule whose redox-responsive behavior complements the design of our tumor microenvironment-responsive nanopolymer for targeted colon cancer therapy.^5,6^ Baicalein exhibits strong cytotoxic effects across a range of cancer cell lines, notably those derived from colon, liver and breast cancers. This compound shows significant potential in inhibiting the growth and survival of these malignant cells.^4^ Reported IC_50_ values range between 20 and 200 µM depending on the cell line, with significant antiproliferative effects observed in human colon carcinoma HCT-116 cells.^7^ Mechanistically, baicalein induces apoptosis and also inhibits angiogenesis and metastasis by regulating cell cycle progression through modulation of pathways involved in cellular signalling, including PI3K/Akt, MAPK, and NF-κB.^5,8^ Moreover, its capacity to overcome multidrug resistance underscores its suitability for combination therapies.^8^

Despite these promising properties, baicalein faces pharmacokinetic limitations including poor water solubility, low bioavailability, rapid metabolism, and limited intestinal absorption, which collectively hinder its clinical translation.^5^ To address these issues, nanocarrier-based delivery systems have emerged as a viable solution to enhance stability, solubility and targeted delivery of baicalein to tumor locations. Nanotechnology offers a transformative platform capable of improving drug pharmacokinetics, biodistribution, and tumor selectivity while minimizing systemic toxicity.^9^ Various nanocarriers including liposomes, micelles, dendrimers, and polymeric nanoparticles have been explored for drug delivery, among which human serum albumin (HSA)-based nanoparticles have attracted particular attention.^10^

HSA naturally occurring plasma protein (65 kDa), plays an essential physiological role in maintaining osmotic balance and transporting endogenous and exogenous molecules.^11^ Its inherent properties—such as high biocompatibility, biodegradability, non-immunogenicity, and strong binding affinity—make it a superior carrier for nanoparticle formulation.^12^ HSA contains multiple binding sites, including cysteine and lysine residues that allow both covalent and non-covalent interactions with hydrophobic molecules like baicalein.^13^ Moreover, HSA nanoparticles exhibit enhanced tumor accumulation *via* receptor-mediated endocytosis and the enhanced permeability and retention (EPR) effect, an important feature of tumor vasculature that allows macromolecules to preferentially accumulate within tumor tissues.^14^ The therapeutic success of albumin-based nanoparticles is exemplified by nab-paclitaxel (Abraxane®), an FDA-approved formulation, which validates the therapeutic feasibility of this carrier system.^12,14^ Compared to free drugs, HSA nanoparticles provide enhanced solubility, controlled release, and prolonged circulation, thereby maintaining therapeutic drug concentrations and reducing systemic side effects.

To further extend the systemic circulation and evade immune clearance, HSA nanoparticles can be surface-modified with polyethylene glycol (PEG) concluded a process known as PEGylation. PEG, a hydrophilic and biocompatible polymer approved by the FDA, forms a steric hydration shell around nanoparticles, preventing protein adsorption and recognition by the mononuclear phagocyte system.^15^ PEGylation significantly improves pharmacokinetics by reducing opsonization, prolonging circulation half-life, and enhancing tumor accumulation through the EPR effect.^16^ Moreover, PEG modification minimizes immunogenicity and systemic toxicity, enabling sustained and controlled drug release. PEGylated formulations of chemotherapeutics have demonstrated enhanced solubility, extended plasma retention, and improved therapeutic index compared to their non-PEGylated counterparts.^16^ Therefore, PEGylated HSA nanoparticles represent an ideal delivery platform for improving the anticancer efficacy of baicalein.

Beyond conventional nanoparticle design, metabolic targeting has emerged as a complementary strategy for selective cancer therapy. In CRC, dysregulation of the arginine metabolism pathway is a hallmark of tumor progression.^17^ Arginine, a semi-essential amino acid, acting as vital characters in nitric oxide synthesis, cell proliferation and polyamine biosynthesis also. CRC cells exhibit enhanced arginine uptake and metabolism driven by overexpression of enzymes such as endothelial nitric oxide synthase (eNOS), argininosuccinate synthetase (ASS1), and ornithine decarboxylase (ODC), which collectively promote tumor growth, angiogenesis, and immune escape.^18,19^ Arginine transporters particularly cationic amino acid transporter 1 (CAT-1, SLC7A1) and solute carrier family 6 member 14 (SLC6A14) are upregulated in CRC cells, facilitating continuous arginine influx and metabolic reprogramming.^20^ Interestingly, l-citrulline, a by-product of arginine metabolism, utilizes the same transporters for cellular entry.^21^ Once inside the cell, citrulline can be converted back to arginine *via* the argininosuccinate pathway, sustaining nitric oxide production and pro-oncogenic signaling. These findings suggest that citrulline and its transporters can be strategically exploited for targeted drug delivery or metabolic interference in CRC.^22^

Based on this rationale, the present study emphases on the development of baicalein-loaded human serum albumin nanoparticles surface-modified with polyethylene glycol and functionalized with l-citrulline (HSA-BA@PEG-LC NPs) for the treatment of colorectal cancer. The design aims to enhance the bioavailability, solubility, and tumor-targeting potential of baicalein while utilizing citrulline-mediated uptake pathways to improve selective accumulation in CRC cells. This multifunctional nanoplatform integrates natural compound pharmacology, nanotechnology, and metabolic targeting to achieve synergistic therapeutic benefits. By combining PEG-induced stealth characteristics with citrulline-based tumor selectivity, HSA-BA@PEG-LC nanoformulation have expected to provide superior anticancer efficacy, reduced systemic toxicity, and enhanced tumor specificity, offering a promising platform for advanced colorectal cancer therapy.

## 2 Materials and methods

### 2.1 Materials

Baicalein (BA) (5,6,7-trihydroxyflavone, purity ≥98%), human serum albumin (HSA, Fraction V, high purity), and *O*-(2-aminoethyl) polyethylene glycol (NH_2_-PEG) were obtained from Sigma Aldrich Co., LLC (USA). l-Citrulline (LC), Streptomycin and penicillin, were also obtained from the same supplier. Fetal bovine serum (FBS) was purchased from GIBCO. Dimethyl sulfoxide (DMSO), Rhodamine 123, and 3-(4,5-dimethyl-2-thiazolyl)-2,5-diphenyl-tetrazolium bromide (MTT) reagents were procured from Himedia (India). All other analytical-grade chemicals were provided by Merck Ltd and SRL Pvt. Ltd, Mumbai. Deionized and ultrapure water was prepared using a Milli-Q purification system (Millipore Corporation, Billerica, MA, USA).

### 2.2 Cell lines

The human colon cancer cell line HCT-116 was obtained from IMGENEX INDIA (Bhubaneswar, Odisha, 751024, India). The cells were cultured in Dulbecco's Modified Eagle Medium (DMEM) improved with 10% fetal bovine serum (FBS) and an antibiotic–antimycotic solution having with penicillin (100 U mL^−1^), streptomycin (10 mg mL^−1^), and l-glutamine (4 mM). The cultures were kept at 37 °C in a humidified atmosphere of 95% relative humidity and 5% CO_2_ to confirm optimum cell growth. For experimental processes, around 1 × 10^6^ viable cells per milliliter were used. Subculturing was carried out by detaching adherent cells with trypsin-EDTA, followed by the addition of fresh medium when the cultures reached around 70% confluence to sustain active proliferation.

### 2.3 Isolation and cultivation of human lymphocyte cells (HLCs)

Human lymphocyte cells (HLCs) were obtained from peripheral blood samples collected from young volunteers those who are healthy, following the method outlined by Hudson and Hay.^23^ Briefly, 4–5 mL of whole blood was gently layered onto an equal volume of Histopaque 1077 (Sigma-Aldrich, USA) and centrifuged for 30 minutes at 2000 rpm on normal room temperature. The subsequent lymphocyte layer was carefully collected and washed three times with phosphate-buffered saline (PBS, pH 7.4) to eliminate any remaining plasma and platelets. The isolated HLCs were then suspended in RPMI-1640 medium having 10% fetal bovine serum (FBS) and cultivated with 5% CO_2_ and humidified incubator at 37 °C for 24 hours prior to cytotoxicity evaluation.

### 2.4 *In silico* docking analysis of baicalein and l-citrulline

Molecular docking was done to predict binding interactions and free binding energies (Δ*G*) of BA and LC with selected target proteins using AutoDock Vina^24^ integrated within UCSF Chimera.^25^ The crystallographic structures of the target proteins were saved from the RCSB Protein Data Bank (http://www.rcsb.org/pdb): Hsp90 Chaperone Inhibitor (PDB ID: 2VCJ),^26^ human serum albumin (HSA) (PDB ID: 1AO6), and LC with cationic amino acid transporter 1 (PDB ID: 8WNT). Briefly, charges were allocated using the ANTECHAMBER algorithm,^27^ and minimization of the system's energy was carried out with Swiss-PdbViewer (SPDBV).^28^ Ligand structures were initially drawn in ChemDraw, converted to SDF format using Chem3D, and subsequently transformed into PDB format *via* Chimera. Gasteiger charges were applied for docking.^29^ Docking poses and interactions were visualized using Discovery Studio Visualizer.

### 2.5 ADME prediction

*In silico* ADME profiling of BA and LC was performed using SwissADME^30^ to predict their physicochemical and pharmacokinetic properties. Parameters assessed included water solubility, lipophilicity (log *P*), bioavailability radar (oral drug-likeness evaluation), drug-likeness rules, and pharmacokinetics. These analyses provided insights into the oral bioavailability and drug-likeness of the studied ligands.

### 2.6 Synthesis of HSA-BA@PEG-LC NPs

#### 2.6.1 Preparation of HSA nanoparticles (HSA NPs)

HSA nanoparticles were prepared using a modified desolvation method aimed at improving yield and stability. In brief, 175 mg of HSA was dissolved into 8 mL of 0.5% (v/v) aqueous acetic acid under continuous vortex agitation. Dropwise ethanol (20 mL) was then added at a controlled rate of 1 mL min^−1^ while maintaining constant stirring at 37 °C and 1000 rpm. The gradual addition of ethanol brought about the appearance of a turbid suspension, indicating nanoparticle formation. The reaction mixture was further stirred for 24 h to ensure complete nanoparticle assembly. Subsequently, ethanol was removed by vacuum distillation, and the protein matrix was cross-linked using 8% glutaraldehyde (0.5 mL per mg of HSA). The obtained nanoparticles were redisposed in 10 mL of deionized water, washed thrice with ultrapure water, and finally collected by centrifugation at 16 000 rpm to eliminate residual reactants.^31^

#### 2.6.2 Preparation of baicalein-loaded HSA nanoparticles (HSA-BA NPs)

For drug encapsulation, a BA stock solution (0.1 mg mL^−1^) was pre-mixed with the HSA solution prior to ethanol addition.^31,32^ The subsequent desolvation and cross-linking steps were identical to those described for the blank HSA nanoparticles. The obtained HSA-BA nanoparticles were thoroughly washed and collected under the same conditions to ensure uniformity in particle characteristics.

#### 2.6.3 Synthesis of l-citrulline conjugated PEG (PEG-LC)

LC was coupled to amino-terminated polyethylene glycol (NH_2_-PEG) *via* carbodiimide-mediated conjugation. Briefly, 70.6 mg of LC was solubilized in 10 mL of a 1 : 1 (v/v) DMSO–Milli-Q water mixture, and the pH of the solution was adjusted to 8.0 using dilute NaOH. To activate the carboxyl groups of LC, EDC (65.92 mg) and NHS (0.32 mmol) were added consecutively under gentle stirring at room temperature. After 4 hours of activation, 50 mg of NH_2_-PEG (pre-dispersed in water) was slowly introduced into the chemical mixture. The coupling reaction was continued overnight with constant stirring.^33^ The resulting PEG-LC conjugate was collected and lyophilized for subsequent applications.^34^

#### 2.6.4 Surface PEGylation of HSA-BA NPs and HSA-BA@PEG-LC NPs formulation

**Fig. 1:**
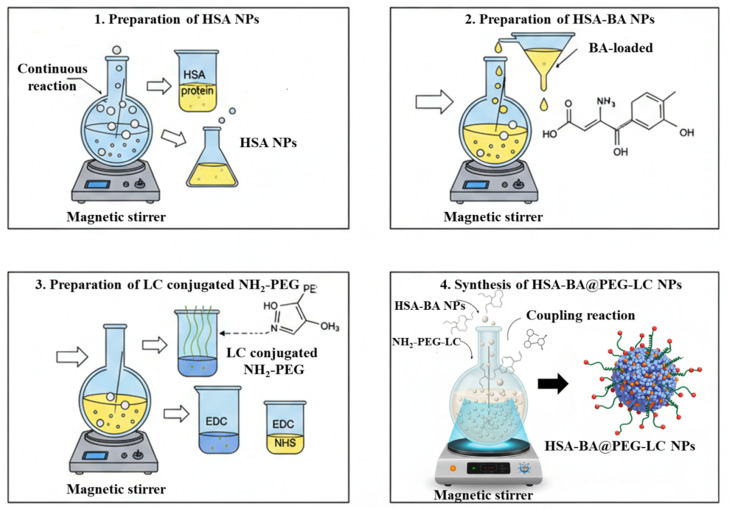
Schematic illustration of the synthesis and surface functionalization of HSA-BA@PEG-LC NPs, showing baicalein encapsulation within HSA and PEG-LC conjugation on the nanoparticle surface.

The surface of HSA-BA nanoparticles was modified with PEG-LC through the creation of amide bonds between the carboxyl groups of PEG-LC and the amino groups on albumin. To begin, PEG-LC (20 mg) was dissolved in 1 mL of ultrapure water and continuously stirred at 500 rpm under ambient conditions. Activation of the PEG-LC carboxyl groups was achieved by adding EDC (0.5 mmol) and NHS (0.5 mmol), each prepared in 750 µL of PBS (pH 7.4), and the mixture was incubated for 30 minutes to allow the reaction to proceed. The pH of the activated solution was then adjusted to 7.0 using 2 M NaOH or 2 M HCl. Following this, an aqueous suspension of HSA-BA nanoparticles (10 mg in 2.5 mL of distilled water) was slowly introduced dropwise into the activated PEG-LC solution. The combined mixture was stirred continuously for 4 hours under dark conditions to minimize oxidative degradation. After the reaction, excess or unbound PEG-LC was eliminated through centrifugation at 10 000 rpm for 30 minutes, followed by thorough washing with distilled water. The nanoparticles were then purified by dialysis using a 12 kDa molecular weight cutoff membrane against 100 mL of PBS (pH 7.4) at 37 °C for 2 hours to remove any residual reactants. Finally, the resulting nanoparticle dispersion was filtered through a 0.2 µm hydrophilic syringe filter, stored at −20 °C and subsequently lyophilized for 24 hours to obtain the dry HSA-BA@PEG-LC NPs (Fig. 1).

### 2.7 Characterization of HSA-BA@PEG-LC NPs

The HSA NPs, HSA-BA NPs, and HSA-BA@PEG-LC NPs were characterized using various analytical techniques, including dynamic light scattering (DLS), zeta potential measurement, UV-visible spectroscopy, Fourier-transform infrared (FTIR) spectroscopy, and scanning electron microscopy (SEM).

#### 2.7.1 Evaluation of particle size distribution and zeta potential

The hydrodynamic size, polydispersity index (PDI), and surface charge (zeta potential) of HSA nanoparticles and HSA-BA@PEG-LC NPs were determined using DLS on a Zetasizer Nano ZS instrument (ZEN 3600, Malvern Instruments, UK). All experiments were repeated three times to confirm measurement reliability. The PDI values were employed to assess the uniformity of nanoparticle dispersion, while the zeta potential values were analyzed to understand colloidal stability and the electrostatic characteristics of the particle surfaces.^35^

#### 2.7.2 UV-visible spectroscopic analysis

Lyophilized HSA NPs, HSA-BA NPs, and HSA-BA@PEG-LC NPs were reconstituted in deionized water *via* mild sonication, and their absorption spectra were recounted over the 200–800 nm wavelength range using a Shimadzu UV-245 double-beam spectrophotometer (Japan).^36^

#### 2.7.3 FTIR spectroscopy analysis

FTIR analysis was performed to identify characteristic functional groups and verify molecular interactions among the HSA, BA, PEG, and LC components. The spectra were acquired using a Nicolet 6700 FTIR spectrometer (Thermo Fisher Scientific, USA) over the wavenumber range of 4000–500 cm^−1^. The samples were finely ground with spectroscopic-grade KBr and compressed into transparent pellets prior to scanning. Comparative spectral analysis provided evidence of bond formation and conformational changes associated with nanoparticle synthesis.^37^

#### 2.7.4 Morphology assessment by scanning electron microscopy (SEM)

The morphological characteristics and surface topology of synthesized HSA-BA@PEG-LC NPs were examined by scanning electron microscopy (CAMSCAN-2, JEOL, Japan) operated at an accelerating voltage of 15 kV. Dried nanoparticle samples were fixed onto copper grids coated with a thin layer of carbon and affixed to aluminum stubs using conductive carbon adhesive. A thin gold film was sputter-coated onto the samples using a JEOL Auto Fine Coater (JEOL1600) to improve conductivity. SEM imaging provided detailed visualization of particle size, shape, and surface architecture.^35^

#### 2.7.5 Nuclear magnetic resonance (NMR) spectroscopy

To further confirm the structural composition and molecular conjugation, ^1^H NMR spectroscopy was performed on freeze-dried HSA-BA@PEG-LC NPs dissolved in deuterated chloroform (CDCl_3_). The spectra were measured using a Bruker Avance III spectrometer operating at 400 MHz. The chemical shifts corresponding to characteristic proton environments were analysed to validate successful conjugation and confirm the integrity of the nanoparticle formulation.^38^

### 2.8 Study of encapsulation efficiency and drug loading

The concentration of BA was obtained through the calibration curve. The curve was constructed by measuring the absorbance of known BA concentrations (1–1000 µg mL^−1^) at *λ*_max 270 nm using a UV-Vis spectrophotometer (Shimadzu UV-245, Japan), yielding the regression equation: *y* = 0.0028*x* + 0.1598 *R*^2^ = 0.9915. A 10 mg sample of the nanoparticle formulation was suspended in an aqueous medium and centrifuged at 15 000 rpm for 30 minutes to remove any unencapsulated BA from the nanoparticle fraction. The amount of free BA in the supernatant was determined from the calibration curve, and encapsulation efficiency (EE) and loading capacity (LC) were calculated accordingly.^39^1
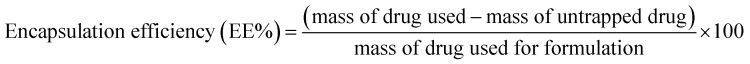
2
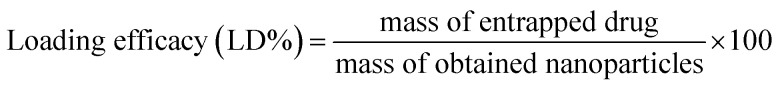


### 2.9 *In vitro* drug release study

The *in vitro* release behavior of BA from HSA-BA@PEG-LC NPs was evaluated using the dialysis technique. The nanoparticle suspensions were sealed within dialysis membranes and immersed in two different release media: acetate-buffered saline (ABS, pH 5.0) and PBS, pH 7.4. Both media were supplemented with 2% fetal bovine serum (FBS) to mimic the acidic tumor microenvironment and normal physiological conditions, respectively. The systems were maintained at 37 °C under continuous shaking at 120 rpm. At predetermined time intervals, aliquots of the release medium were collected, and the concentration of BA released was determined by UV-Vis spectrophotometry at 276 nm. This experiment was performed in triplicate (*n* = 3), and resulting cumulative release profiles were analysed to investigate the pH-dependent release characteristics of the HSA-BA@PEG-LC NPs.^39^

### 2.10 Analysis of *in vitro* cellular uptake of HSA-BA@PEG-LC NPs

#### 2.10.1 Time-dependent cellular uptake of BA and confocal study of drug uptake

The cellular uptake of free BA, HSA-BA NPs, and HSA-BA@PEG-LC NPs was evaluated in HCT-116 cells using combined spectrophotometric and fluorescence approaches. For quantitative evaluation, 5 × 10^5^ cells per well were plated in 24-well culture plates and left to adhere for 24 hours. The cells were then exposed to 0.1 mg mL^−1^ of BA or the respective nanoparticle formulations for incubation periods ranging from 1 to 24 hours. At each time point, cells were washed, trypsinized, lysed by probe sonication, and centrifuged. Intracellular BA levels were determined spectrophotometrically at 205 nm using a calibration curve, with untreated cells as blank. For qualitative assessment, BA and HSA-BA@PEG-LC NPs were tagged with Rhodamine B (0.05%) and incubated on HCT-116 cells grown on coverslips at IC50 doses for 4 h. After fixation with 4% paraformaldehyde and DAPI nuclear staining, confocal microscopy revealed intracellular localization. This integrated approach demonstrated enhanced cellular internalization of HSA-BA@PEG-LC NPs, supporting their potential as an efficient targeted drug delivery system.^39^

### 2.11 *In vitro* cytotoxicity assays

The cytotoxic activity was investigated using the MTT assay on HCT-116 colon cancer cells and normal human lymphocyte cells (HLCs). Cells were seeded in 96-well plates and exposed to varying concentrations (10–100 µg mL^−1^) of BA, HSA NPs, HSA-BA NPs, and HSA-BA@PEG-LC NPs for 24 hours under standard culture conditions (37 °C, 5% CO_2_). Following incubation, the cells were washed and exposed to the MTT reagent, allowing viable cells to convert it into purple formazan crystals. Subsequently, the crystals were dissolved in DMSO, and the absorbance was quantified at 540 nm using a microplate reader to determine cell viability. To examine the time-dependent cytotoxic behavior, an additional set of experiments was performed at 24- and 48-hour intervals. All results were expressed as the mean ± SD from three independent experimental replicates.^35^3
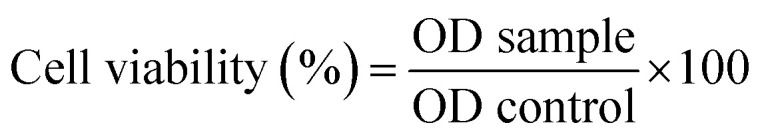


The concentration required for 50% cytotoxicity (IC_50_) was determined graphically using GraphPad Prism software.

### 2.12 Immunofluorescence staining of the actin cytoskeleton

The morphological alterations and structural reorganization of the F-actin cytoskeleton in HCT-116 cells after treatment with different nanoparticle formulations were examined using immunofluorescence staining with phalloidin and DAPI. Briefly, the treated cells were washed twice with PBS, pH 7.4 and fixed in 2.5% glutaraldehyde for 15 minutes at room temperature. The fixed cells were then treated with 0.5% Triton X-100 in PBS for 5 minutes to permeabilize the cells to facilitate the penetration of fluorescent probes. Actin filaments were stained with phalloidin (100 nM) for 30 minutes, followed by nuclear counterstaining with DAPI (100 nM) at 37 °C for 15 minutes. After thorough PBS washing to remove residual dyes, the samples were mounted using an antifade mounting medium. Confocal laser scanning microscopy was employed to capture high-resolution fluorescence images, enabling the evaluation of actin filament distribution, cytoskeletal organization, and morphological modifications induced by nanoparticle exposure.^39^

### 2.13 Intracellular ROS and GSH/GSSG analysis

HCT-116 cells underwent treatment with BA, HSA-BA nanoparticles, and HSA-BA@PEG-LC NPs at a 5 µg per mL concentration for 24 hours to assess oxidative responses. Following exposure, the cells were rinsed with phosphate buffer and stained with 1 µg per mL H_2_DCFDA for 30 minutes at 37 °C to detect intracellular ROS through DCF fluorescence imaging using a ZEISS fluorescence microscope. To evaluate redox status, cells were lysed, and proteins were precipitated using 4% sulfosalicylic acid. The resulting supernatant was reacted with 0.6 mM DTNB for spectrophotometric quantification of reduced glutathione (GSH) at 412–420 nm, normalized to total protein (µg mg^−1^). For oxidized glutathione (GSSG) determination, samples were pretreated with 2-vinylpyridine before DTNB reaction. All experiments were performed thrice to ensure data reliability and reproducibility.^37^

### 2.14 Assessment of mitochondrial membrane potential (Δ*Ψ*_m_)

The influence of BA and HSA-BA@PEG-LC NPs on the mitochondrial membrane potential of HCT-116 cells were assessed after a 24-hour treatment at their respective IC_50_ levels. Following exposure, cells were washed thrice with cold PBS and stained with Rhodamine 123 (1.5 µM) for 15 minutes at 37 °C. Fluorescence imaging using a LEICA DFC295 microscope (green filter) was then conducted to examine mitochondrial health. The findings revealed that HSA-BA@PEG-LC nanoparticles caused a more pronounced loss of mitochondrial membrane potential compared to free BA, suggesting that these nanoparticles enhance apoptosis by promoting mitochondrial dysfunction.^40^

### 2.15 Assessment of nuclear morphological changes

Early apoptotic changes in nuclei were examined using DAPI and RNase-propidium iodide (PI) staining. HCT-116 cells (1 × 10^6^ mL^−1^) were exposed to BA and HSA-BA@PEG-LC NPs at concentrations corresponding to their respective IC_50_ values for 24 hours. Fluorescence microscopy (NIKON ECLIPSE LV100POL) was used to observe chromatin condensation and alterations in nuclear morphology. Compared with free BA, HSA-BA@PEG-LC NPs caused more distinct nuclear condensation and higher PI uptake, indicating a stronger induction of apoptosis.^38,40^

### 2.16 Flow cytometry analysis of cell cycle regulation

HCT-116 cells (2 × 10^5^ per well) were seeded in 6-well plates and cultured at 37 °C in a humidified environment with 5% CO_2_ for 24 hours. After this incubation, the cells were treated with HSA-BA@PEG-LC nanoparticles at their respective IC_50_ concentrations and maintained for another 24 hours. After treatment, cells were isolated *via* centrifugation at 1200 rpm for 10 minutes, washed with PBS, and fixed with 70% chilled ethanol. The fixed cell pellets were stored at −20 °C until analysis. For cell-cycle assessment, the samples were washed thrice with PBS, permeabilized using 0.1% Triton X-100, and treated with RNase A (200 µg mL^−1^) at 37 °C for 30 minutes. DNA was then stained with propidium iodide (100 µg mL^−1^) for 15 minutes, followed by flow cytometric evaluation of cell-cycle distribution.^39,40^

### 2.17 Apoptotic cell analysis using annexin V-FITC/propidium iodide

For assessment of apoptosis, HCT-116 cells were treated with HSA-BA@PEG-LC NPs at their IC_50_ concentration for 24 hours under identical experimental conditions. After incubation, the cells were harvested, rinsed twice with ice-cold PBS and subsequently resuspended in annexin-binding buffer. To quantify different cell populations, 5 µL of Annexin V-FITC and 5 µL of PI were sequentially added for dual staining. The samples were incubated in the dark for 15 minutes at room temperature to prevent photobleaching. Finally, the stained cells were analyzed using flow cytometry, and obtained fluorescence data were processed with dedicated software to assess the proportion of viable, early apoptotic, late apoptotic, and necrotic cells, enabling precise evaluation of nanoparticle-induced apoptosis.^41^

### 2.18 DNA fragmentation by alkaline comet assay

DNA fragmentation in HCT-116 cells was determined through a modified alkaline comet assay to evaluate genotoxic responses. Cells (1 × 10^6^ mL^−1^) were treated with BA and HSA-BA@PEG-LC NPs at their respective IC_50_ concentrations for 24 hours. After incubation, cells were collected, rinsed twice with PBS, and embedded in 0.7% low-melting agarose layered on pre-coated 1% agarose slides. The slides were then lysed in a chilled buffer containing 2.5 M NaCl, 0.1 M EDTA, 0.01 M Tris, and 0.01% Triton X-100 (pH 10) for 20 minutes. Electrophoresis was performed at 80 mA for 30 minutes in an alkaline NaOH–EDTA buffer. Subsequently, slides were neutralized for 10 minutes, stained with ethidium bromide (0.01 mg mL^−1^), and examined using a fluorescence microscope (LEICA DFC295, Germany).^39,42^ DNA damage was quantified using the damage index (DI):

4 Total comet score or damage index (DI) = (0 × *N*_0_) + (1 × *N*_1_) + (2 × *N*_2_) + (3 × *N*_3_) + (4 × *N*_4_)

where *N*_0_ – intact cells without a tail; *N*_1_ – cells with a tail shorter than the head diameter; *N*_2_ – cells with a tail measuring one to two times the head diameter; *N*_3_ – cells with a tail longer than twice the head diameter; and *N*_4_ – cells consisting almost entirely of a tail. Fluorescence intensity, tail length, and head area were measured using ImageJ software, analyzing 100 randomly selected cells per group for reproducibility.

### 2.19 Antioxidant assay methods

#### 2.19.1 DPPH radical scavenging assay

The antioxidant activity of HSA, HSA-BA NPs, and HSA-BA@PEG-LC NPs was evaluated through the DPPH (2,2-diphenyl-1-picrylhydrazyl) radical scavenging assay, adapted from the procedure of Roy *et al.* (2022) with minor modifications.^43^ Briefly, varying concentrations of each sample (50–200 µg mL^−1^) were combined with an equal volume of DPPH solution (0.1 mM) prepared using methanol. The resulting mixtures were kept in the dark at room temperature for 30 minutes to avoid photodegradation of the radical. After incubation, absorbance was determined at 517 nm using a UV-Vis spectrophotometer. The decrease in absorbance indicated the free radical scavenging efficiency of each formulation, reflecting their potential antioxidant capacity relative to the native and modified nanoparticle systems. The radical scavenging activity was expressed as percentage inhibition, calculated according to the formula:

5 Inhibition (%) = (*A*_0_ − *A*_s_)/*A*_0_ × 100

where *A*_0_ represents the absorbance of the control and *A*_s_ represents the absorbance of the sample.

#### 2.19.2 Nitric oxide (NO) radical scavenging assay

The NO scavenging activity was evaluated using a modified procedure of Can *et al.* (2022).^44^ Various concentrations (50–200 µg mL^−1^) of HSA, HSA-BA NPs, and HSA-BA@PEG-LC NPs were combined with 10 mM sodium nitroprusside solution in phosphate-buffered saline (PBS, pH 7.4). The mixtures were exposed to light and incubated at 25 °C for 150 minutes to generate nitric oxide. After incubation, equal volumes of Griess reagent containing 1% sulfanilamide, 0.1% naphthylethylenediamine dihydrochloride, and 2% phosphoric acid were added and allowed to stand for 10 minutes at room temperature, forming a pink azo compound. The absorbance of this product was recorded at 546 nm using a UV-Vis spectrophotometer to determine the NO radical scavenging potential.

#### 2.19.3 Superoxide radical scavenging assay

The superoxide anion scavenging activity was determined using a modified method based on the protocol described by Chun *et al.* (2003).^45^ The assay mixture contained 468 µM nicotinamide adenine dinucleotide (NADH), 156 µM nitroblue tetrazolium (NBT), and 60 µM phenazine methosulfate (PMS) prepared in 100 mM phosphate buffer with a pH of 7.4. To initiate the reaction, different concentrations (50–200 µg mL^−1^) of HSA, HSA-BA NPs, and HSA-BA@PEG-LC NPs were mixed to the solution. The obtained mixture kept at room temperature for 5 minutes to facilitate the reaction. The absorbance corresponding to the formation of formazan from the reduction of NBT by superoxide radicals was measured at 560 nm using a UV-Vis spectrophotometer to evaluate antioxidative potential.

#### 2.19.4 Lipid peroxidation inhibition assay

A reaction system was formulated by mixing 0.16 mM FeSO_4_, 30 mM KCl, and 0.06 mM ascorbic acid with varying concentrations (50–500 µg mL^−1^) of HSA, HSA-BA NPs, or HSA-BA@PEG-LC NPs. The prepared mixtures were incubated at 37 °C for 1 hour to induce oxidative activity. Post-incubation, 1.5 mL of 1% (w/v) thiobarbituric acid and 1.0 mL of 10% (w/v) trichloroacetic acid were added, and the reaction volume was adjusted to 4 mL with distilled water. The samples were then heated in a water bath at 95 °C for 30 minutes to allow formation of the malondialdehyde–thiobarbituric acid (MDA–TBA) adduct, followed by cooling to room temperature. To extract the chromogenic complex, 1 mL of distilled water and 5 mL of an *n*-butanol: pyridine mixture (15 : 1, v/v) were added. The resulting mixtures were centrifuged at 4000 rpm for 10 minutes, and the upper organic phase was collected. The concentration of the MDA–TBA complex was quantified spectrophotometrically by recording the absorbance at 532 nm.^45^

### 2.20 Estimation of cellular area and fluorescence intensity

Fluorescence micrographs of the cultured cells were acquired using a fluorescence microscope (LEICA DFC295, Germany) and subsequently processed with ImageJ software (NIH, Bethesda, MD, USA). Images were initially converted to 8-bit grayscale, followed by threshold adjustment to obtain binary representations that maximized visualization of the cell attachment area. The cell area was quantified from the generated binary masks using the “Analyze Particles” tool. Fluorescence intensity was determined by measuring the mean gray values within the defined cellular regions of interest (ROIs).

### 2.21 Statistical analysis

All experiments were conducted in a minimum of thrice independent replicates. Data are expressed as the mean ± standard deviation. Statistical analysis between the control and treatment groups was performed using one-way analysis of variance (ANOVA) with GraphPad Prism software (version 8.5; GraphPad Software, San Diego, CA, USA). A *p*-value of less than 0.05 was considered to indicate statistical significance.

## 3 Result and discussion

### 3.1 Molecular docking analysis

**Fig. 2:**
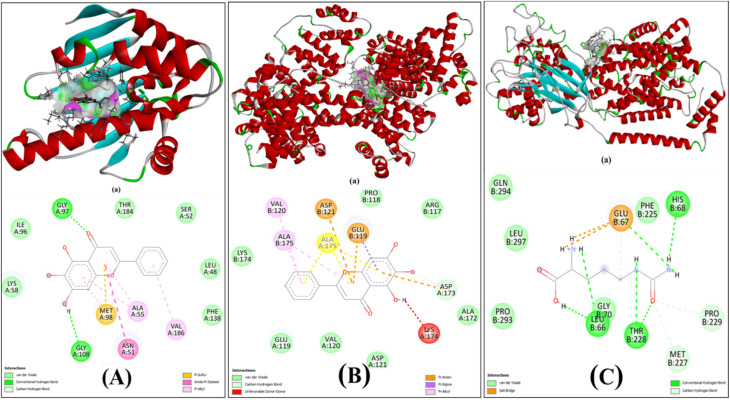
Molecular docking studies. (A) Baicalein with Hsp90 inhibitors (PDB: 2VCJ): 3D view shows binding in the active site; 2D diagram highlights hydrogen bonds (GLY97, GLY108), pi-sulfur/alkyl, amide-π stacking, and van der Waals interactions. (B) Baicalein with HSA: 3D binding in α-helices/β-strands; 2D diagram shows pi-anion, pi-Sigma/Alkyl, carbon–hydrogen bonds, and van der Waals contacts. (C) l-Citrulline with cationic amino acid transporter 1 (PDB: 8WNT): 3D and 2D views show hydrogen bonds, salt bridges, van der Waals interactions; chemical structure included.

Molecular docking analysis showed strong binding affinities of BA and LC with their respective target proteins (Fig. 2). The design shows free energy change of ligand BA for interactions with protein 2VCJ, Δ*G* = −8.2 kcal mol^−1^ (Table 1) inside a grid box of 28.6 × 12.7 × 19.82 Å with dimensions 31 × 31 × 31 Å along three axes (Fig. 2A). The 2D diagram (Fig. 2B) shows that ligand BA forms two hydrogen bonding interactions with 97th Glycine residue and 108th aspartic acid of the A chain in the binding site of protein 2VCJ. The active pocket through hydrogen bonds (GLY97, GLY108), carbon hydrogen bonding and pi-sulfur interaction (MET98), pi-alkyl interactions (ALA55, ASN51, VAL186), and amide-π stacking (ALA55), further stabilized by van der Waals contacts (LYS58, ILE96, THR184) (Fig. 2A). Similarly, BA demonstrated high affinity toward HSA, forming pi-anion (GLU119, ASP121), pi-Sigma/Alkyl (ALA175, VAL120), and carbon–hydrogen bonding (ALA175), along with van der Waals interactions, suggesting its efficient transport and favourable pharmacokinetics. As HSA is being used for the formation of carrier nanoparticles for the ligand the compatibility of binding to any site of HSA becomes essential to understand, for that we have performed docking for any site covering entire protein inside the grid. The data reveal the free energy change of ligand BA for the interactions with protein 1AO6, Δ*G* = −8.5 kcal mol^−1^ (Table 2) inside a grid box covering entire protein (Fig. 2B). In parallel, LC showed stable docking within the Cationic amino acid transporter 1 (PDB ID: 8WNT), mediated by hydrogen bonds (GLU67, HIS68, THR228, GLY70), carbon hydrogen bonds, van der Waals interactions and salt bridges highlighting its potential modulatory role (Fig. 2C). Collectively, these results demonstrate strong and specific molecular interactions, supporting therapeutic relevance of BA as an anticancer agent and LC as a promising adjunct in modulating cancer-related pathways (Table 1).

**Table 1:** Molecular docking analysis of baicalein and l-citrulline with target proteins

Target protein (PDB ID)	Ligand	Docking score (kcal mol^−1^)
2VCJ	Baicalein	−8.2
1AO6	Baicalein	−8.5
8WNT	l-Citrulline	−5.4

**Table 2:** Physicochemical characterization of HSA-BA nanoparticles and HSA-BA@PEG-LC nanoparticles, including particle size, zeta potential, polydispersity index (PDI), encapsulation efficiency (EE%), and loading capacity (LC%)

Group	Particle diameter (nm)	Zeta potential (mV)	Polydispersity index (PDI)	Encapsulation efficiency (EE%)	Loading capacity (LC%)
HSA-BA NPs	186.14 ± 4.74	−14.54 ± 0.28	0.231 ± 0.02	63.341 ± 1.43	8.875 ± 0.326
HSA-BA@PEG-LC NPs	232.52 ± 5.31	−27.21 ± 0.32	0.319 ± 0.04	86.621 ± 2.83	13.352 ± 0.145

### 3.2 Prediction of oral bioavailability

**Fig. 3:**
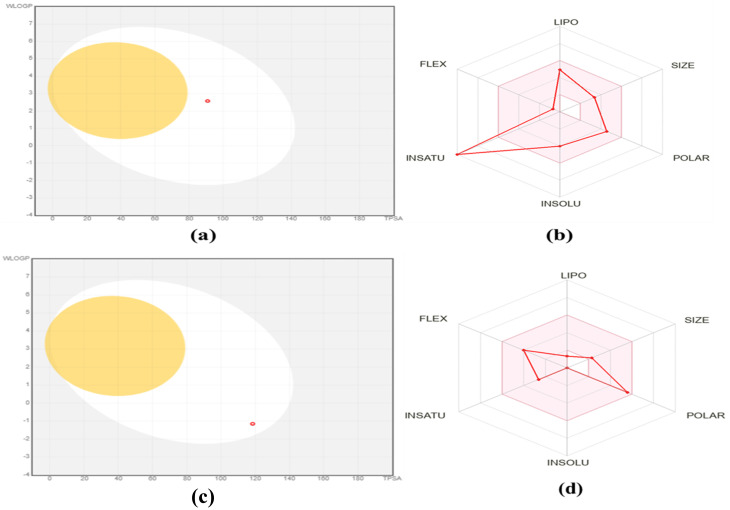
Boiled egg diagram (a) and (b) bio-radar of BA for oral bio-availability and boiled egg diagram (c) and (d) bio-radar profile of LC for oral bioavailability assessment.

The BOILED-Egg diagram visualizes the position of BA (red dot) within the optimal physicochemical space for gastrointestinal absorption (white region) and blood–brain barrier penetration (yellow region). The *x*-axis represents topological polar surface area (TPSA), and the *y*-axis represents lipophilicity (WLOGP). Baicalein within the white region suggests good passive gastrointestinal absorption but poor blood–brain barrier permeability. The bio-radar (bioavailability radar plot) displays six key drug-likeness parameters: lipophilicity (LIPO), size, polarity (POLAR), solubility (INSOLU), saturation (INSATU), and flexibility (FLEX). The red polygon indicates BA profile in comparison to the optimal physicochemical space (pink area). BA shows acceptable lipophilicity and polarity but deviates in flexibility and saturation, which may slightly limit its oral bioavailability (Fig. 3A and B). *In silico* prediction revealed limited oral bioavailability of LC; however, its docking studies demonstrated stable interactions with the active site of Wnt/β-catenin protein, a key regulator in colon cancer progression. LC formed hydrogen bonds, van der Waals and salt bridges contacts with critical residues, suggesting its potential role in modulating Wnt/β-catenin signaling. These molecular interactions indicate that LC may exert anticancer effects locally within the colon microenvironment (Fig. 3C and D).

### 3.3 Characterization of HSA-BA@PEG-LC NPs

The HSA-BA@PEG-LC NPs were characterized for their physicochemical properties using DLS to define particle size and polydispersity, UV-Vis spectroscopy to confirm nanoparticle, scanning electron microscopy (SEM) for morphological analysis, FTIR to identify functional group interactions, and NMR to verify the chemical structure and successful conjugation of HSA, BA, and PEG-LC. These analyses collectively confirmed the uniform size, spherical morphology, and effective incorporation of BA within the HSA-PEG-LC matrix.

#### 3.3.1 Hydrodynamic size analysis by DLS

**Fig. 4:**
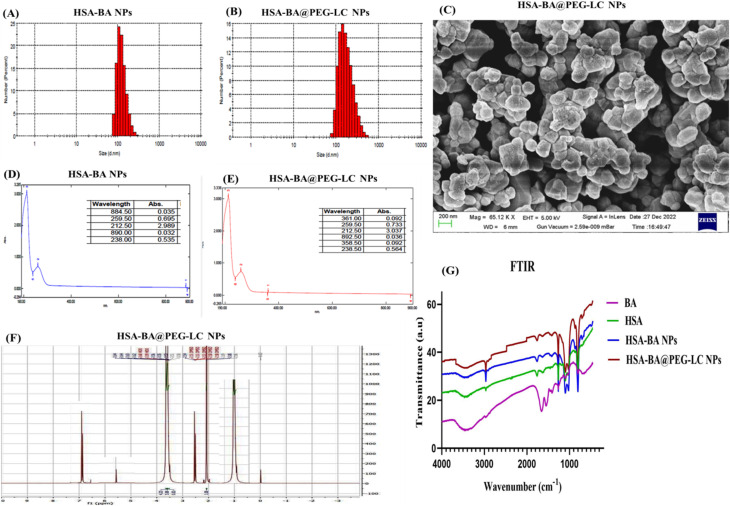
Physicochemical characterization of HSA-BA NPs and HSA-BA@PEG-LC NPs. (A) DLS profile of HSA-BA NPs showing narrow distribution (B) DLS profile of HSA-BA@PEG-LC NPs. (C) SEM image HSA-BA@PEG-LC NPs. (D) UV-Vis absorption spectrum of HSA-BA NPs. (E) UV-Vis absorption spectrum of HSA-BA@PEG-LC NPs. (F) NMR spectra indicating characteristic chemical shifts of HSA-BA@PEG-LC NPs. (G) FTIR spectra showing functional group interactions confirming successful encapsulation and surface modification of HSA-BA@PEG-LC NPs.

DLS determined hydrodynamic size distribution of nanoparticles.^46^ HSA-BA NPs exhibited a relatively narrow size distribution with an average diameter of 186.14 ± 4.74 nm (Fig. 4A), whereas HSA-BA@PEG-LC NPs displayed a slightly increased size (232.52 ± 5.31 nm) (Fig. 4B), indicating successful PEGylation and l-citrulline conjugation on nanoparticle surface. The narrow polydispersity index (PDI < 0.3) in both cases suggests good colloidal stability and uniformity. Zeta potential of HSA-BA NPs was −14.54 ± 0.28 mV and −27.21 ± 0.32 mV of HSA-BA@PEG-LC NPs, indicating enhanced surface charge and improved colloidal stability also.

#### 3.3.2 UV-visible spectroscopy for optical properties of nanoparticles

The UV-Vis absorption spectra revealed distinct absorbance peaks corresponding to BA-loaded nanoparticles. HSA-BA NPs showed characteristic absorption maxima around 212 nm and 238 nm, which are attributable to the π → π* transitions of aromatic rings and n → π* transitions of carbonyl groups in BA (Fig. 4D). After PEG-LC modification (Fig. 4E), slight peak shifts and changes in absorbance intensity were observed, confirming successful conjugation and surface modification. The additional absorbance at 361 nm can be attributed to the interaction between HSA-BA@PEG-LC NPs, which alters the local electronic environment.^47^

#### 3.3.3 Morphological evaluation of nanoparticles through SEM

SEM analysis demonstrated that both HSA-BA and HSA-BA@PEG-LC NPs were predominantly spherical in morphology with smooth surfaces (Fig. 4C). The uniform and non-aggregated distribution suggests efficient encapsulation and stabilization of BA within the HSA matrix. PEG and LC coating produced slightly larger and denser structures, which corroborates the DLS findings. The nanoscale dimensions further support their potential for cellular uptake and systemic circulation.^48^

#### 3.3.4 Molecular interactions and surface functionalization of HSA-BA@PEG-LC NPs in FTIR

The FTIR spectra (Fig. 4G) clearly illustrate structural changes associated with BA encapsulation into HSA and subsequent PEG-LC surface functionalization. The spectrum of free BA displayed its characteristic vibrational bands, including a broad peak at 3410 cm^−1^ corresponding to phenolic O–H stretching, a strong band at 1650 cm^−1^ attributed to flavone carbonyl (C=O) stretching, and a peak near 1510 cm^−1^ related to aromatic C=C stretching, confirming the presence of the BA flavonoid backbone. Native HSA exhibited prominent amide I (1655 cm^−1^, C=O stretching) and amide II (1540 cm^−1^, N–H bending/C–N stretching) peaks, which are typical markers of protein secondary structure. Upon encapsulation of BA into HSA, the amide I and II bands showed slight shifts and changes in intensity, indicating noncovalent interactions—particularly hydrogen bonding and hydrophobic associations—between BA and the HSA matrix. These spectral modifications confirm successful incorporation of BA within the protein environment. In the FTIR spectrum of HSA-BA@PEG-LC NPs, additional peaks appeared at 2880–2920 cm^−1^ corresponding to C–H stretching from PEG methylene groups, along with a distinct band at 1635 cm^−1^ associated with the N–H bending vibration of the guanidinium moiety of l-citrulline. The presence of these new bands, coupled with shifts in amide regions, demonstrates that PEG and LC are covalently and/or stably attached to the nanoparticle surface. Collectively, the FTIR spectra of free components *versus* encapsulated and functionalized formulations confirm successful BA loading into HSA and effective PEG-LC conjugation, validating the structural integrity and surface modification of the engineered nanopolymer.

#### 3.3.5 Molecular-level validation of conjugation of HSA-BA@PEG-LC NPs using NMR

The ^1^H NMR spectra offered molecular-level confirmation of BA encapsulation and PEG-LC modification (Fig. 4F). Free BA displayed well-defined aromatic proton resonances between *δ* 6.20–7.80 ppm, corresponding to its A- and B-ring protons, along with downfield hydroxyl proton signals above *δ* 9.0 ppm. In the HSA-BA nanoparticle spectrum, these aromatic signals were broadened and reduced in intensity, reflecting restricted mobility due to hydrophobic and hydrogen-bonding interactions with the HSA protein scaffold. Native HSA exhibited typical aliphatic proton signals in the *δ* 0.8–1.5 ppm region, as well as backbone amide and α-proton signals between *δ* 3.2–4.5 ppm. Upon PEGylation, characteristic resonances at *δ* 3.35 ppm (–CH_2_ protons of PEG repeating units) were clearly evident, confirming surface grafting of PEG chains. Additionally, in HSA-BA@PEG-LC NPs, new multiple signals appeared at *δ* 1.6–1.8 ppm and *δ* 3.0–3.2 ppm, which resemble to the methylene and methine protons of LC. The presence of these distinct resonances, coupled with the disappearance of free BA hydroxyl proton peaks, strongly indicates that BA is encapsulated within the protein core, while PEG and LC are chemically conjugated with nanoparticle surface. Together, NMR data provide convincing molecular conjugation for the successful fabrication of a multifunctional nanoparticle system in which drug encapsulation and surface functionalization are simultaneously achieved.

### 3.4 Encapsulation efficiency (EE%), loading capacity (LC%), and yield of HSA-BA@PEG-LC NPs

The EE%, LC% and yield of HSA-BA@PEG-LC NPs are summarized in Table 2. HSA-BA NPs exhibited an EE of 63.34 ± 1.43% and LC of 8.87 ± 0.32%, with a formulation yield of 72%. In contrast, HSA-BA@PEG-LC NPs demonstrated significantly improved encapsulation (86.62 ± 2.83%) and loading capacity (13.35 ± 0.14%), accompanied by a higher yield of 88%. The improvement in EE and LC upon PEG-LC modification suggests enhanced drug–polymer interactions and greater stabilization of BA within the nanoparticle matrix. The higher yield of HSA-BA@PEG-LC NPs further indicates efficient nanoparticle recovery and minimal drug loss during formulation. Together, these findings confirm that PEG-LC functionalization not only enhances drug encapsulation and loading but also improves overall process efficiency, making the formulation more suitable for large-scale and reproducible drug delivery applications.

### 3.5 *In vitro* pH-responsive drug release profile of HSA-BA@PEG-LC NPs

**Fig. 5:**
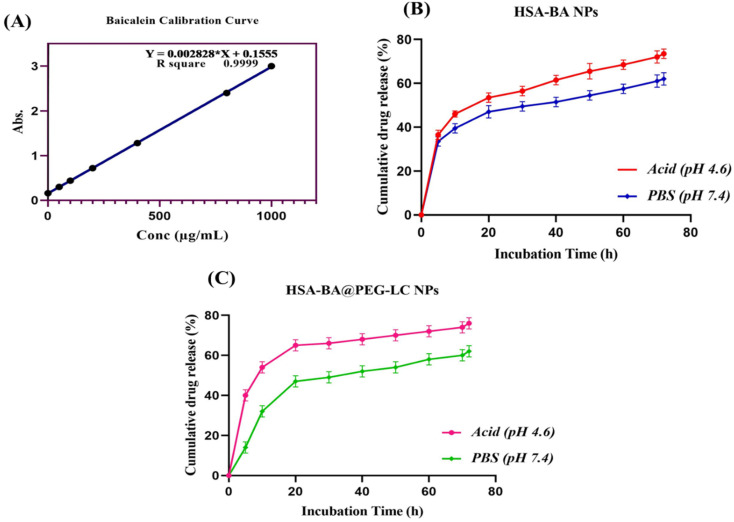
(A) Calibration curve of baicalein (B) cumulative release profile of baicalein from HSA-BA NPs at acidic pH (4.6) and physiological pH (7.4) over 72 h, indicating higher release under acidic conditions. (C) Cumulative release profile of baicalein from HSA-BA@PEG-LC NPs at acidic pH (4.6) and physiological pH (7.4), demonstrating improved sustained and pH-responsive release in PEG-LC modified nanoparticles. Data are presented as mean ± SD.

*In vitro* drug release studies of HSA-BA@PEG-LC NPs were determined using the standard calibration curve (Fig. 5A). Drug release profiles (Fig. 5B and C) revealed pH-dependent behaviour. HSA-BA NPs showed an initial burst release (40% within 10 h), followed by sustained release, reaching 68% at pH 4.6 and 58% at pH 7.4 after 72 h. In contrast, HSA-BA@PEG-LC NPs displayed higher stability and controlled release, with 82% at pH 4.6 and 52% at pH 7.4. The biphasic release pattern indicated effective encapsulation and improved LC, with PEG-LC modification enhancing acidic pH responsiveness. These results demonstrate that PEG-LC functionalization enhances both stability and targeted release, making HSA-BA@PEG-LC NPs promising candidates for tumor-targeted drug delivery.

### 3.6 Study of *in vitro* cellular uptake of HSA-BA@PEG-LC NPs

#### 3.6.1 Time-dependent cellular uptake by spectrophotometric analysis

**Fig. 6:**
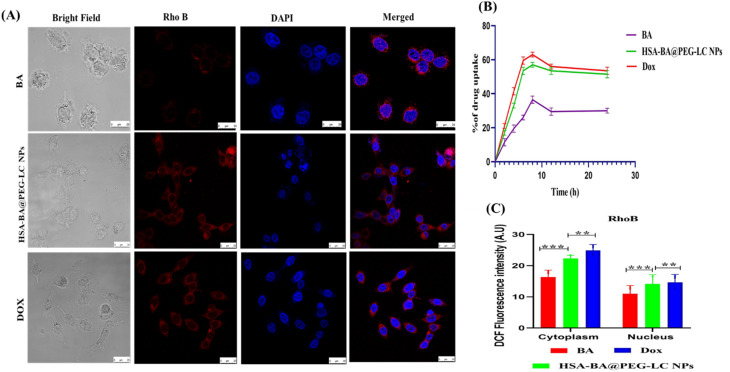
Time-dependent cellular uptake and intracellular localization of baicalein formulations. (A) Confocal microscopy images of cells treated with BA, HSA-BA@PEG-LC NPs, and DOX. (B) Spectrophotometric analysis of drug uptake over 24 h showing higher uptake efficiency for HSA-BA@PEG-LC NPs compared to free BA, with DOX used as a positive control. (C) DCF fluorescence intensity in the cytoplasm and nucleus of HCT-116 cells after treatment with BA, Dox, or HSA-BA@PEG-LC NPs. Data are represented as mean ± SD.

The spectrophotometric drug uptake study revealed a distinct time-dependent internalization profile for BA, HSA-BA@PEG-LC NPs, and doxorubicin (DOX) (Fig. 6B). Free BA exhibited comparatively low cellular uptake, reaching a maximum of 35% after 8 h and then plateauing. In contrast, HSA-BA@PEG-LC NPs showed significantly higher uptake, peaking at 55% within 8 h and maintaining stability over 24 h. DOX, treated as a positive control, confirmed the highest uptake (62%) at 8 h, followed by a gradual decline. These findings confirm that PEG-LC modification markedly improves nanoparticle-mediated delivery and cellular retention of BA compared to its free form.

#### 3.6.2 Intracellular localization by confocal microscopy

Confocal fluorescence microscopy further validated nanoparticle internalization (Fig. 6A). Free BA showed weak red fluorescence, indicating limited intracellular accumulation. HSA-BA@PEG-LC NPs exhibited intense and evenly distributed red fluorescence within the cytoplasm, while DOX-treated cells displayed strong nuclear and cytoplasmic localization, consistent with its known mechanism of action. The co-localization of red fluorescence (drug signal) with blue nuclear staining (DAPI) confirmed successful intracellular trafficking. These results collectively demonstrate that HSA-BA@PEG-LC NPs enhance cellular uptake and facilitate efficient intracellular delivery of BA, making them superior to free BA in targeted delivery applications.^49^

### 3.7 MTT assay in HCT-116 cells

**Fig. 7:**
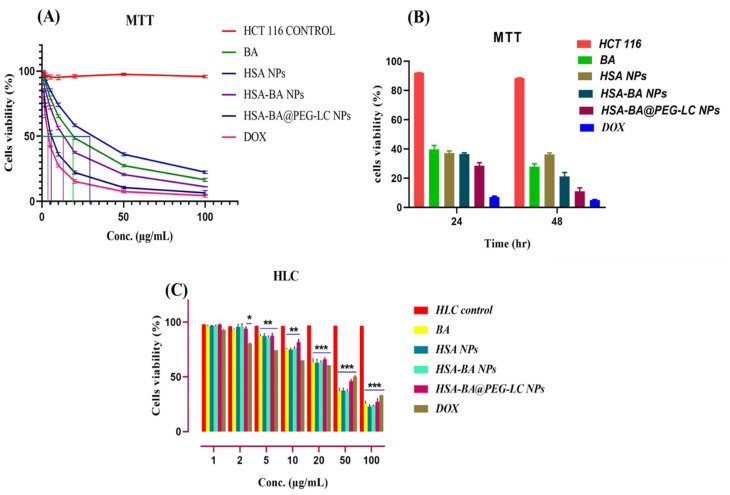
Cytotoxicity of BA and its nanoparticle formulations in HCT-116 cells and human lymphocytes (HLC). (A) Dose-dependent cell viability of HCT-116 cells treated with BA, HSA NPs, HSA-BA NPs, HSA-BA@PEG-LC NPs, and DOX after 48 h, determined by MTT assay. (B) Time-dependent cytotoxicity of HCT-116 cells treated with the same formulations at 24 and 48 h. (C) Cytotoxicity in normal human lymphocytes (HLC) showing low toxicity of all formulations, with HSA-based nanoparticles maintaining >60% viability across all tested concentrations. Data represent mean ± SD; *p < 0.05, **p < 0.01, ***p < 0.001 *versus* control.

The cytotoxic effects of BA, HSA NPs, HSA-BA NPs, and HSA-BA@PEG-LC NPs on HCT-116 cells were evaluated using a concentration-dependent MTT assay (Fig. 7A). Cell viability decreased progressively with increasing concentrations of all formulations. Among the tested groups, HSA-BA@PEG-LC NPs exhibited the highest cytotoxicity, followed by HSA-BA NPs, BA alone, and HSA NPs. The calculated IC_50_ values after 48 h treatment were as follows: BA (18.29 µg mL^−1^), HSA NPs (27.34 µg mL^−1^), HSA-BA NPs (12.37 µg mL^−1^), and HSA-BA@PEG-LC NPs (5.42 µg mL^−1^). These results indicate a substantial enhancement of cytotoxic activity upon nanoformulation, particularly with PEGylated l-citrulline functionalization. Doxorubicin (positive control) demonstrated expected high cytotoxicity, confirming the assay validity, with cell viability decreasing to 3.5% at 100 µg mL^−1^. At lower concentrations (1–10 µg mL^−1^), HSA-BA@PEG-LC NPs reduced cell viability to 84.42–35.15%, whereas BA alone maintained relatively higher cell survival (94.82–64.65%). At higher concentrations (50–100 µg mL^−1^), HSA-BA@PEG-LC NPs exhibited significant cytotoxicity, reducing cell viability to 9.78–5.13%, indicating a strong dose-dependent effect. In comparison, free BA and HSA-BA NPs showed moderate cytotoxicity, highlighting the role of PEG-LC modification in enhancing intracellular drug delivery and potency.

#### 3.7.1 Time-dependent cytotoxicity

The time-dependent MTT assay further revealed that HSA-BA@PEG-LC NPs caused a more pronounced reduction in cell viability at 48 hours compared to 24 hours, indicating enhanced cellular internalization and prolonged drug action (Fig. 7B). This highlights the advantage of PEGylated nanocarriers in maintaining therapeutic efficacy over time.

#### 3.7.2 Cytotoxicity in human lymphocytes (HLCs)

Assessment of cytotoxicity in normal HLCs indicated that all treatment groups exhibited minimal toxicity across a wide range of concentrations (1–100 µg mL^−1^). Even at higher doses, cell viability remained above 60–70% for nanoparticle formulations, while DOX showed moderate toxicity (Fig. 7C). This suggests that HSA-based BA formulations, particularly HSA-BA@PEG-LC NPs, possess selective cytotoxicity toward cancer cells while sparing normal lymphocytes, underscoring their potential safety and biocompatibility for therapeutic applications.

### 3.8 Elevated ROS generation induces redox imbalance

**Fig. 8:**
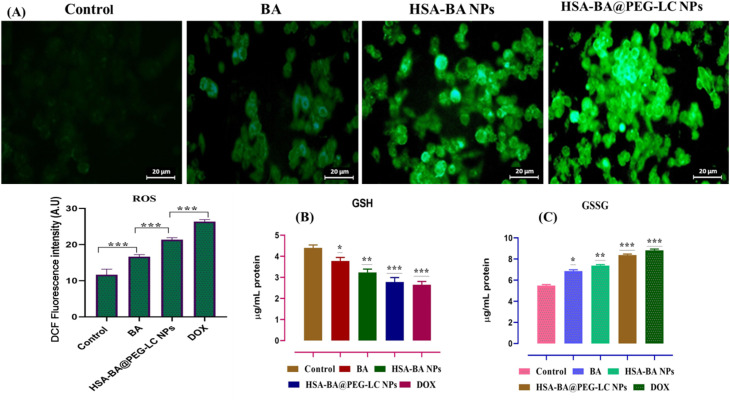
(A) Fluorescence micrographs showing ROS generation of BA, HSA-BA NPs, and HSA-BA@PEG-LC NP-treated cells and quantitative analysis of ROS (DCF fluorescence intensity). (B) and (C) Glutathione (GSH), and oxidized glutathione (GSSG) data represent mean ± SD; *p < 0.05, **p < 0.01, ***p < 0.001 *versus* control.

Treatment of cells with BA, HSA-BA NPs, HSA-BA@PEG-LC NPs, and DOX resulted in a significant and progressive increase in ROS levels as detected by H_2_DCF staining and quantified by DCF fluorescence intensity (Fig. 8A). Notably, HSA-BA@PEG-LC NPs exhibited the most pronounced ROS induction, closely followed by DOX, with both showing higher oxidative stress compared to BA and HSA-BA NPs treatments. Robust ROS generation correlates with increased cellular stress and potential for enhanced anticancer activity due to oxidative damage.^39^

### 3.9 GSH depletion and GSSG accumulation reflect redox status

Parallel measurement of glutathione metabolic parameters revealed a marked decrease in reduced GSH along with an elevation in oxidized GSSG levels in treatment groups compared to control (Fig. 8B and C). The decrease in GSH and rise in GSSG was most significant in the HSA-BA@PEG-LC NPs and DOX groups, indicating that increased ROS surpassed cellular antioxidant capacity, promoting GSH oxidation and redox imbalance. This depletion of GSH and accumulation of GSSG further validates the observation of high oxidative stress, and suggests impaired detoxification capability and increased pro-apoptotic signaling in treated cells. Elevated ROS, depleted GSH, and increased GSSG indicate that treatments disrupt redox balance, with excessive ROS oxidizing GSH to GSSG and impairing cellular pathways. HSA-BA@PEG-LC NPs caused the strongest effect, demonstrating their potent redox-modulating and anticancer activity.^37^

### 3.10 Chromatin condensation and cell death indicated by DAPI and PI staining

**Fig. 9:**
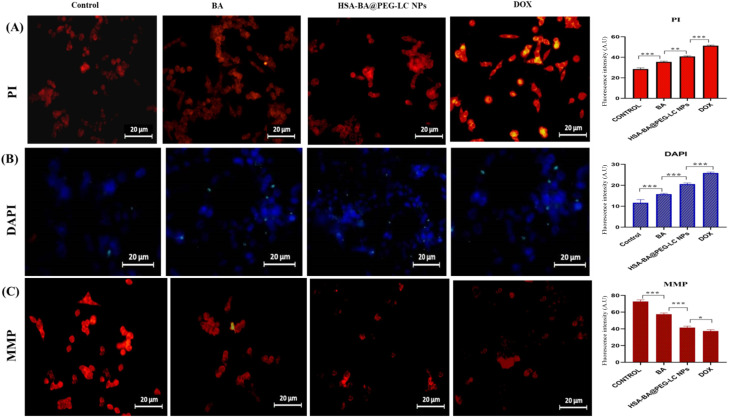
Apoptosis analysis by fluorescence microscopy. (A) DAPI: chromatin condensation; (B) PI: increased membrane permeability; (C) MMP (Rhodamine 123): mitochondrial depolarization in cells treated with BA, HSA-BA@PEG-LC NPs, and DOX.

Treatment with BA, HSA-BA@PEG-LC NPs, and DOX induced significant chromatin condensation as evidenced by increased DAPI fluorescence intensity relative to control cells (Fig. 9B). Concurrently, PI staining demonstrated elevated membrane permeability, a hallmark of late apoptosis or necrosis, with progressively higher PI fluorescence in treated groups, especially notable in DOX and HSA-BA@PEG-LC NP treatments (Fig. 9A). The condensed chromatin and enhanced PI uptake indicate effective induction of apoptotic cell death *via* nuclear DNA fragmentation and compromised membrane integrity by the tested compounds, confirming their potent cytotoxic effect.^50^

### 3.11 Mitochondrial membrane potential (MMP) disruption

MMP analysis revealed a marked decrease in fluorescence intensity in BA, HSA-BA@PEG-LC NPs, and DOX-treated cells compared to control, indicative of mitochondrial depolarization (Fig. 9C). The loss of MMP, most pronounced with HSA-BA@PEG-LC NPs and DOX, suggests mitochondrial dysfunction, a critical step in the intrinsic apoptosis pathway. This collapse of MMP confirms the involvement of mitochondrial-mediated apoptosis, complementing chromatin condensation and membrane permeability data to delineate the mechanisms underlying treatment-induced cell death.^51^

### 3.12 Treatment-dependent cytoskeletal disassembly

**Fig. 10:**
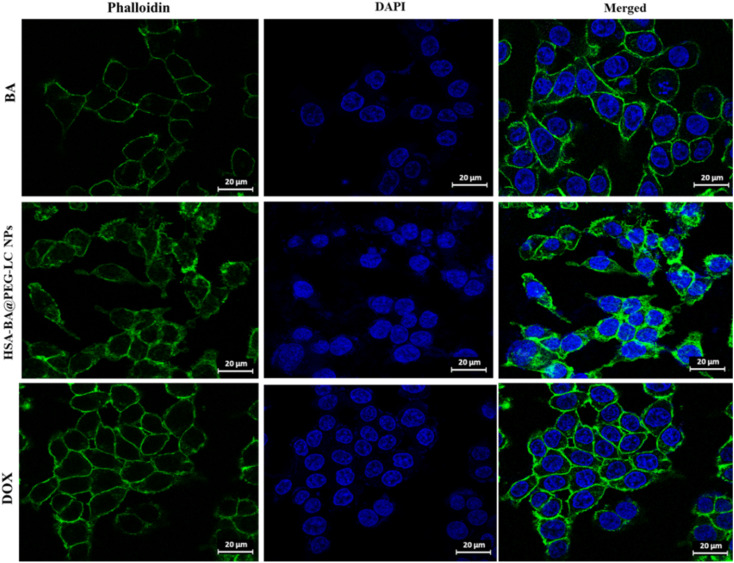
Confocal microscopy images of HCT 116 cells treated with BA, HSA-BA@PEG-LC NPs, and DOX. Cells were stained with phalloidin and DAPI.

Confocal microscopy analysis was performed on HCT 116 cells following treatment with BA, HSA-BA@PEG-LC NPs, and DOX to investigate cytoskeletal organization (Fig. 10). Phalloidin staining revealed the structure of F-actin filaments (green), while DAPI highlighted cell nuclei (blue). HCT 116 cells exposed to HSA-BA@PEG-LC NPs demonstrated pronounced disruption and fragmentation of actin filaments compared to cells treated with BA and DOX. The merged images clearly showed significant cytoskeletal disassembly in the HSA-BA@PEG-LC NPs group, indicating compromised cellular integrity and suggesting enhanced cytotoxic or apoptotic effects relative to BA and DOX treatments. In contrast, BA and DOX treated cells retained more continuous and intact cytoskeletal architecture. These observations suggest that HSA-BA@PEG-LC NPs may offer superior anti-cancer potential in HCT 116 cells by promoting greater cytoskeletal disruption, as revealed by confocal imaging.^39,52^

### 3.13 Cell cycle arrest analysis by flow cytometry

**Fig. 11:**
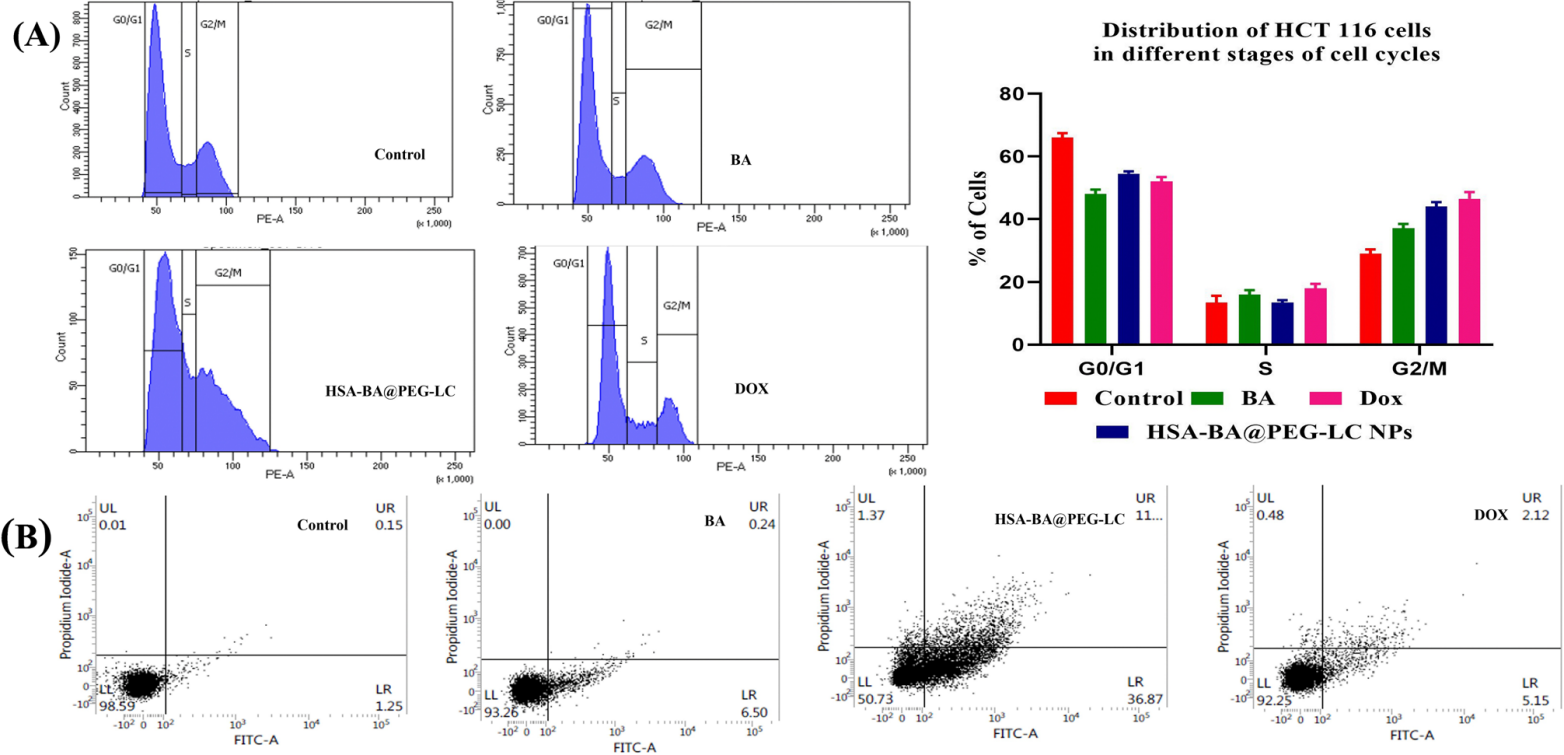
(A) Representative flow cytometry histograms showing the distribution of HCT 116 cells across G0/G1, S, and G2/M phases after treatment with Control, BA, DOX, and HSA-BA@PEG-LC NPs. (B) Annexin V/PI double staining dot plots present the percentage of viable, early apoptotic, late apoptotic, and necrotic HCT 116 cells under respective treatments.

Flow cytometric analysis (Fig. 11A) of HCT 116 cells revealed significant alterations in cell cycle distribution upon treatment with BA, DOX, and HSA-BA@PEG-LC NPs compared to the control group. The histogram profiles demonstrate distinct inhabitants in G0/G1, S, and G2/M phases.^53^ Treatment with BA and DOX resulted in pronounced accumulation of cells in the G2/M phase (45–50%), indicative of cell cycle arrest at this checkpoint. In contrast, HSA-BA@PEG-LC NPs treatment induced a marked increase in the G0/G1 phase (61%), signifying arrest at this earlier phase of the cell cycle. The reduction in the S phase population further substantiates the efficiency of these treatments in halting cellular proliferation *via* cell cycle modulation.^54,55^

### 3.14 Apoptosis induction by annexin V/PI double staining

Annexin V/PI staining provided robust evidence of apoptosis induction after various treatments.^56^ In the control group, 98% of the cells were viable, with negligible apoptotic populations. BA and DOX treatments significantly increased the fraction of early and late apoptotic cells, as indicated by the upper right and lower right quadrants, respectively. Notably, HSA-BA@PEG-LC NPs achieved superior induction of apoptosis, with the highest percentage of cells undergoing programmed cell death, early apoptosis around 6.5% and late apoptosis over 5%. These findings confirm the enhanced pro-apoptotic effect of nanoparticle-based delivery systems compared to conventional treatments (Fig. 11B).

### 3.15 DNA damage assessment by comet assay

**Fig. 12:**
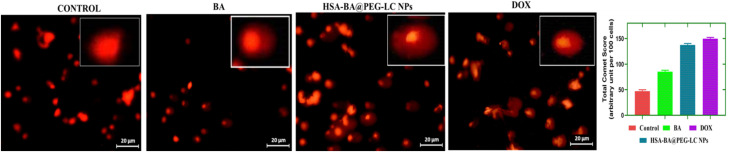
Representative fluorescence micrographs of comet assay in HCT 116 cells exposed to Control, BA, DOX, and HSA-BA@PEG-LC nanoparticles.

The comet assay results (Fig. 12) demonstrate significant DNA damage in HCT 116 cells following various treatments, as visualized by the increased comet tail lengths and DNA migration away from the nucleus. In the control group, nuclei remained intact with minimal DNA fragmentation, evidenced by compact, rounded comets. BA and DOX treatments induced moderate DNA strand breaks, as reflected by the extended comet tails. Notably, cells treated with HSA-BA@PEG-LC NPs displayed pronounced DNA damage, characterized by distinctly elongated comet tails and diffused DNA, signifying extensive genotoxic effects. The higher degree of DNA fragmentation in the HSA-BA@PEG-LC NPs group confirms that nanoparticle-mediated delivery significantly augments DNA damage compared to free drug treatments, underlining its enhanced therapeutic efficacy in colon cancer cells.^57,58^

### 3.16 Antioxidant assay

#### 3.16.1 DPPH radical scavenging activity

**Fig. 13:**
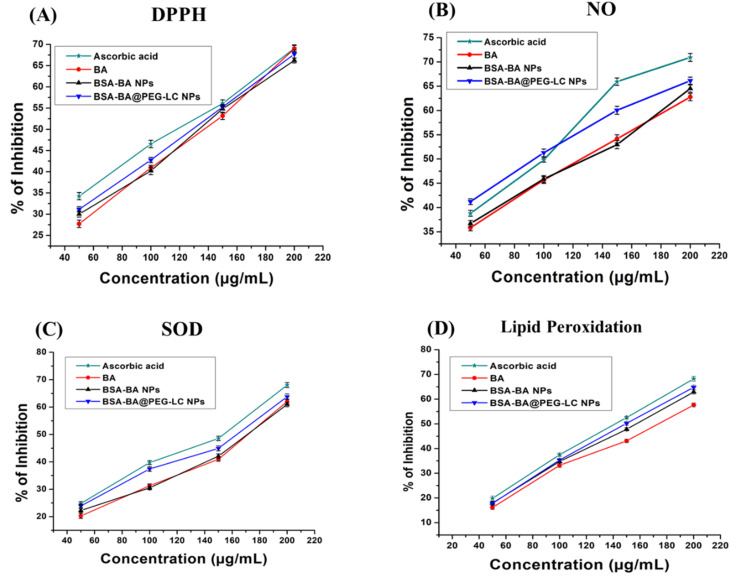
Antioxidant activity of different formulations evaluated by (A) DPPH radical scavenging assay, (B) nitric oxide (NO) radical inhibition assay, (C) superoxide dismutase (SOD)-like activity, and (D) lipid peroxidation inhibition assay. Data represent mean ± SD.

The DPPH assay demonstrated a dose-dependent enhancement in free radical scavenging activity for all tested formulations^43^ (Fig. 13A). The HSA-BA@PEG-LC NPs exhibited significantly improved antioxidant potential with IC_50_ value of 125.46 µg mL^−1^, compared to HSA-BA NPs (138.75 µg mL^−1^) and free BA (145.32 µg mL^−1^). The performance closely approximated that of the standard ascorbic acid (120.19 µg mL^−1^), indicating a synergistic enhancement of antioxidant capacity upon PEG-LC modification. This enhancement can be attributed to the improved solubility, dispersibility, and controlled release characteristics of PEGylated nanoparticles, which increase the effective availability of BA at the reactive interface. Furthermore, the encapsulation within the HSA matrix may protect the active phenolic groups of BA from oxidative degradation, thereby sustaining radical scavenging efficiency.

#### 3.16.2 Nitric oxide (NO) radical inhibition

In the NO radical assay, HSA-BA@PEG-LC NPs showed markedly superior inhibitory effects across all concentrations, with IC_50_ value of 102.29 µg mL^−1^, surpassing both HSA-BA NPs (118.74 µg mL^−1^) and BA (128.42 µg mL^−1^), and approaching the activity of ascorbic acid (94.64 µg mL^−1^). The enhanced inhibition of NO radicals suggests that PEG-LC modification enhances the interaction of nanoparticles with reactive nitrogen species. The improved antioxidant efficacy may stem from increased surface hydrophilicity and better diffusion kinetics of NO toward the antioxidant active sites.^44^ Moreover, the conjugation with PEG-LC likely facilitates the stabilization of BA hydroxyl functionalities, preventing their premature oxidation and enabling more effective scavenging of reactive intermediates (Fig. 13B).

#### 3.16.3 Superoxide (SOD) radical scavenging assay

The SOD assay demonstrated that HSA-BA@PEG-LC NPs possess a stronger superoxide radical scavenging capability with IC_50_ value of 153.57 µg mL^−1^, outperforming HSA-BA NPs (165.21 µg mL^−1^) and BA (172.88 µg mL^−1^). This activity closely parallels that of ascorbic acid (148.17 µg mL^−1^), confirming the retained redox potential of BA upon nanoparticle conjugation (Fig. 13C). The improved SOD-like activity may be linked to the nanoscale organization and PEG-mediated dispersion stability, which enhance electron transfer processes and facilitate the dismutation of superoxide anions.^45^ Additionally, the amphiphilic structure of PEG-LC may promote the accessibility of superoxide radicals toward the active phenolic regions of BA, thus improving the overall scavenging kinetics.

#### 3.16.4 Lipid peroxidation inhibition

The lipid peroxidation assay revealed a notable inhibition pattern consistent with the other antioxidant assays (Fig. 13D). The IC_50_ value of HSA-BA@PEG-LC NPs (159.69 µg mL^−1^) was significantly lower than HSA-BA NPs (171.48 µg mL^−1^) and BA (182.62 µg mL^−1^), and comparable to ascorbic acid (142.67 µg mL^−1^). The superior efficacy of HSA-BA@PEG-LC NPs in suppressing lipid peroxidation may be due to the dual protective effects of HSA and PEG-LC components. The HSA core likely provides a stabilizing microenvironment for BA, while the PEG-LC surface coating enhances lipid membrane interaction and radical trapping efficiency.^43^ These synergistic effects prevent oxidative chain propagation in lipid systems, underlining the formulation potential for mitigating membrane-associated oxidative stress.

## 4 Conclusion

This study introduces HSA-BA@PEG-LC NPs as a robust, multifunctional nanoplatform that integrates targeted delivery, redox modulation, and biocompatible design for colon cancer therapy. By leveraging the strong molecular interactions of BA with Hsp90 and HSA, as well as LC affinity for CAT-1 transporters, the system achieves selective tumor targeting and enhanced intracellular uptake. The nanoparticles demonstrated excellent physicochemical attributes, including uniform nanoscale size, high encapsulation efficiency, and pH-responsive release ideally suited for the acidic tumor microenvironment. *In vitro*, HSA-BA@PEG-LC NPs elicited potent cytotoxic and pro-oxidant effects in HCT-116 cells while sparing normal lymphocytes, underscoring their therapeutic specificity. Mechanistic studies revealed that treatment disrupts redox homeostasis, impairs mitochondrial integrity, and triggers cell-cycle arrest, collectively driving cancer cell death. The pronounced antioxidant capacity further highlights the synergistic role of PEG-LC functionalization and HSA stabilization in sustaining BA bioactivity. Altogether, these findings position HSA-BA@PEG-LC NPs as a promising next-generation nanotherapeutic with strong translational potential for targeted and redox-responsive colon cancer treatment.

## Ethical statement

All experiments were performed in accordance with the “Indian Council of Medical Research (ICMR) ethical guidelines for biomedical research on human participants”, and experiments were approved by the Institutional Ethics Committee of Vidyasagar University, Midnapore (Ref. No. VU/IEC/5/18-24, dated 06.02.24). Informed consents were obtained from human participants of this study.

## Conflicts of interest

The authors declare that they have no conflicts of interest associated with this study.

## Data Availability

All supporting data generated or analysed during this study are presented within the main body of the manuscript. The underlying raw datasets can be obtained from the corresponding author upon reasonable request.
